# Orofacial Complications of the Connective Tissue Disease Systemic Sclerosis

**DOI:** 10.1177/00220345241249408

**Published:** 2024-05-23

**Authors:** M. Sharma, A. Fadl, A. Leask

**Affiliations:** 1 Department of Psychology and Health Studies, University of Saskatchewan, Saskatoon, SK, Canada; 2 College of Dentistry, University of Saskatchewan, Saskatoon, SK, Canada

**Keywords:** interleukin, fibroblast, gingiva, TGF-β, microstomia, fibrosis

## Abstract

Scleroderma (systemic sclerosis, SSc) is an autoimmune fibrosing connective tissue disease of unknown etiology. SSc patients show increased levels of autoantibodies, profibrotic cytokines, and extracellular matrix remodeling enzymes that collectively cause activated (myo)fibroblasts, the effector cell type of fibrosis. Despite these impacts, no disease-modifying therapy exists; individual symptoms are treated on a patient-to-patient basis. SSc research has been principally focused on symptoms observed in the lung and skin. However, SSc patients display significant oral complications that arise due to fibrosis of the not only skin, causing microstomia, but also the gastrointestinal tract, causing acid reflux, and the oral cavity itself, causing xerostomia and gingival recession. Due to these complications, SSc patients have impaired quality of life, including periodontitis, tooth loss, reduced tongue mobility, and malnutrition. Indeed, due to their characteristic oral presentation, SSc patients are often initially diagnosed by dentists. Despite their clinical importance, the oral complications of SSc are severely understudied; high-quality publications on this topic are scant. However, SSc patients with periodontal complications possess increased levels of matrix metalloproteinase-9 and chemokines, such as interleukin-6 and chemokine (C-X-C motif) ligand-4. Although many unsuccessful clinical trials, mainly exploring the antifibrotic effects of anti-inflammatory agents, have been conducted in SSc, none have used oral symptoms, which may be more amenable to anti-inflammatory drugs, as clinical end points. This review summarizes the current state of knowledge regarding oral complications in SSc with the goal of inspiring future research in this extremely important and underinvestigated area.

## What Is Systemic Sclerosis?

Scleroderma (systemic sclerosis, SSc), an autoimmune rheumatic connective tissue disease of unknown etiology, is characterized by a hyperactive immune response and epithelial and vascular damage, which can result in progressive tissue fibrosis affecting not only the skin but also the internal organs and oral cavity (Denton 2015; Distler et al. 2023). The incidence of SSc varies within populations, with the highest occurrence (47/100,000) in Canadian First Nations (Calderon and Pope 2021). SSc is more prevalent in females compared to males (4:1 ratio), with prevalence highest in middle age and in underprivileged households (Calderon and Pope 2021). Juvenile (childhood-onset) SSc is substantially more rare but has similar clinical features to adult SSc (Stevens et al. 2019). SSc does not follow Mendelian inheritance patterns, yet numerous studies have suggested that SSc has an epigenetic component and tends to occur in families that have a history of autoimmune disorders (Zhang et al. 2024). Genetic susceptibility is correlated with alterations in the human leukocyte antigen (HLA) system, which regulates the immune response and antigen presentation to T cells (Machhua et al. 2023).

According to the American College of Rheumatology and European League Against Rheumatism (ACR/EULAR) classification criteria, skin thickening of the fingers extending proximal to the metacarpophalangeal joints is sufficient for a diagnosis of SSc to be made (Hoogen et al. 2013). Alternatively, 7 additional criteria are used: skin thickening of the fingers, fingertip lesions, telangiectasia, abnormal nailfold capillaries, lung involvement, Raynaud’s phenomenon, and the presence of SSc-related autoantibodies. SSc is further divided into 2 classifications based on the degree of organ involvement: limited cutaneous SSc (lcSSc) and diffuse cutaneous SSc (dcSSc) (Herrick et al. 2022). Patients with lcSSc have localized fibrosis restricted to the skin. lcSSc is formerly referred to as “CREST,” which refers to the 5 main features: calcinosis, Raynaud’s phenomenon, esophageal dysmotility, sclerodactyly, and telangiectasia. Conversely, dcSSc patients have progressive thickening of skin and internal organs, including kidneys and lungs, and significantly higher mortality (Herrick et al. 2022). For example, pulmonary fibrosis, the major cause of mortality in dcSSc patients, has a prevalence of ~30% and a 10-y mortality of ~40% (Perelas et al. 2020). The prevalence of lcSSc, dcSSc, and sine scleroderma, in which patients lack skin fibrosis, was recently found to be 64%, 28%, and 8%, respectively (Höppner et al. 2023).

## A Mechanically Stiff, Hypoxic Microenvironment Drives SSc Fibrosis

Although what initiates SSc is unclear, various environmental stimuli such as silica and solvents have been proposed as possible triggers (Barnes and Mayes 2012; Muntyanu et al. 2024). However, regardless of the initiating cause, one of the earliest events that precedes SSc fibrosis is endothelial cell injury and apoptosis causing vascular damage and tissue hypoxia (Suliman and Distler 2013). This hypoxia, however, is insufficient to result in compensatory repair. This vascular damage is presented clinically as Raynaud’s phenomenon and digital ulcers, both of which are common comorbidities of SSc (Suliman and Distler 2013). Vascular damage and hypoxia are also likely to contribute to the myocardial damage and interstitial lung disease/pulmonary arterial hypertension seen in SSc patients (Suliman and Distler 2013; Launay et al. 2017). This hypoxic microenvironment, through the action of hypoxia-inducible transcription factor 1α (HIF-1α), likely directly leads to fibrosis by causing dermal fibroblasts to overproduce extracellular matrix (ECM) components (Distler et al. 2007). Moreover, endothelial (and epithelial) injury, in the context of a heightened immune response, causes the release of fibrogenic chemokines, including transforming growth factor (TGF)-β, endothelin 1 (ET-1), and interleukin (IL) 1a, that promote fibroblasts not only to differentiate into highly contractile myofibroblasts and but also to excessively remodel a collagen-rich ECM (Leask 2009; Aden et al. 2010). This mechanically stiff and hypoxic microenvironment is necessary and sufficient for the initiation, progression, and maintenance of the fibrotic phenotype that severely impairs mobility and organ function (Suliman and Distler 2013; Leask et al. 2023).

The clinical presentation of SSc varies greatly, which further complicates diagnosis. There is no overall disease-modifying treatment; therefore, symptoms are managed depending on individual needs (Lee and Pope 2016). In part due to this complexity, research focused on elucidating either the most debilitating (e.g., Raynaud’s phenomenon, digital ulcers, and cutaneous sclerosis) or life-threatening (e.g., interstitial lung disease, pulmonary arterial hypertension, or renal crisis) symptoms. Intriguingly, oral complications, although they occur early and are easy to identify, are severely understudied (Veale et al. 2016; Gonzalez et al. 2021). Therefore, describing what is known about oral complications in SSc is timely and, accordingly, is the subject of this review.

## Orofacial Implications of SSc

**Figure 1.**
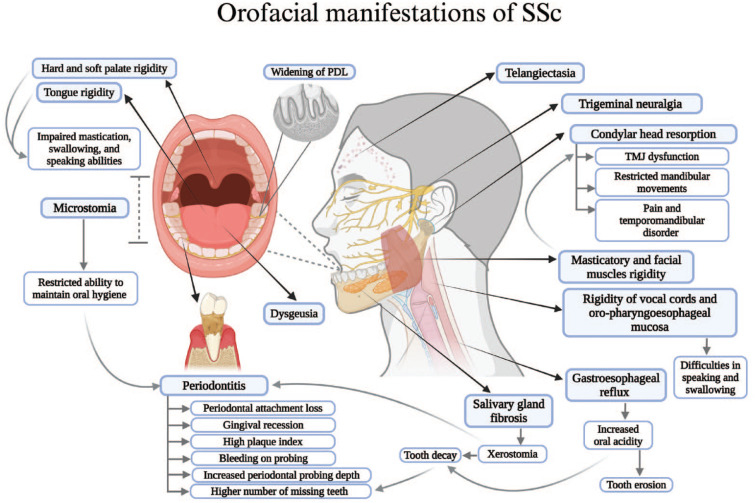
Various orofacial manifestations and complications observed in patients with systemic sclerosis, involving the skin, muscles, salivary glands, temporomandibular joint, mandibular bone, trigeminal nerve, oral mucosa, and periodontium. Figure was created with BioRender.com.

In addition to the significant impact of SSc on internal organ functioning, orofacial manifestations affect up to 80% of SSc patients (Iordache et al. 2019), involving skin, muscles, periodontium, salivary glands, temporomandibular joint (TMJ), trigeminal nerve, and mandibular bone (Fig. 1). However, certain oral complications, such as oral candidiasis in SSc patients undergoing corticosteroid therapy, mouth ulcers secondary to methotrexate, and rare occurrences of calcium channel blocker–induced gingival hyperplasia, may arise as side effects of medications prescribed to alleviate systemic symptoms of SSc (Alantar et al. 2011). Generally, despite their considerable impact on the overall health of SSc patients, orofacial manifestations are often overlooked in favor of addressing more systemic disorders associated with SSc (Veale et al. 2016).

### Facial Skin and Muscle Rigidity

The orofacial involvement of SSc is predominately associated with facial skin thickening and muscle rigidity, resulting in a mask-like face appearance, loss of facial expressions, and a reduction in mouth opening, known as microstomia (Vitali et al. 2010; Gonzalez et al. 2021). Microstomia was observed in 52% to 80% of SSc patients (Gonzalez et al. 2021) and has been related to disease severity (Baron et al. 2015). Microstomia significantly impairs SSc patients’ food intake and promotes the onset of malnutrition by hindering their abilities in mastication and deglutition, adversely impacts SSc patients’ abilities to maintain oral hygiene, and limits the abilities of dental practitioners to provide proper oral examinations or dental treatments (Veale et al. 2016; Almeida et al. 2023). Interestingly, compared to lcSSc patients, microstomia is significantly more evident in dcSSc, contributing to a 2.5 times higher Mouth Handicap in the SSc Scale (MHISS) score, significantly impacting their oral health–related quality of life (OHRQoL) (Jung et al. 2023). Furthermore, fibrosis affecting the orofacial region in SSc may extend to the oropharyngoesophageal mucosa, tongue, and hard and soft palate, causing difficulties in swallowing and speaking and changes in voice (Vitali et al. 2010). These alterations, along with impaired facial expressions, impact SSc patients’ communication abilities and social interaction, causing psychological distress, impairing their quality of life (Gholizadeh et al. 2017).

### Telangiectasia

The appearance of dilated superficial blood vessels, known as telangiectasia, has been observed on the skin, primarily the hand and face, as well as oral mucosa in SSc patients. Such manifestations can serve as indicators of the underlying vascular changes associated with SSc (Crincoli et al. 2016).

### Periodontitis and Gingival Inflammation

SSc patients have more severe periodontitis indicators compared to controls, including periodontal attachment loss, gingival recession, higher plaque and gingival indices, and increased inflammatory biomarkers levels in biofilm and gingival crevicular fluid (GCF) (Buchbender et al. 2021; Jung et al. 2023). However, current data on how periodontal conditions vary among SSc subtypes are limited (Jung et al. 2023). In a study that included lcSSc and dcSSc patients, the frequency of advanced periodontitis (stage III/IV) was higher among the dcSSc group (90%) compared to the lcSSc group (60%) (Stanomir et al. 2023), which resonated with a study that reported increased periodontal attachment loss in dcSSc compared to lcSSc (Pischon et al. 2016). However, exacerbated gingival inflammation has been specifically observed in lcSSc patients (Jung et al. 2023). Conversely, gingival inflammation is not a constant clinical finding in dcSSc patients, where inflammation might be masked by fibrosis, thus complicating the diagnostic process (Jung et al. 2023). However, gingiva in SSc do not exhibit the fibrotic phenotype observed in skin, which might be related to its scar-less healing ability, primarily attributed to the relative resistance of gingival fibroblasts to myofibroblast differentiation and regenerative and fetal-like immunomodulatory properties (Fadl and Leask 2023). Interestingly, widening of the periodontal ligament (PDL) affects more than one-third of SSc patients (Jung et al. 2023). The cause remains unclear, yet excess deposition of collagen within PDL and increased occlusal force secondary to bulky masticatory muscles are commonly implicated (Jung et al. 2023). Collectively, the etiology of the altered periodontal conditions in SSc is unclear and potentially multifactorial. The factors include, xerostomia, restricted ability to maintain oral hygiene due to microstomia, oral microbiome alterations, microangiopathy, immune dysregulation, and pathological ECM remodeling associated with SSc (Baron et al. 2015; Almeida et al. 2023; Jud et al. 2023).

### Salivary Gland Dysfunction

Pathological changes in the salivary glands of SSc patients may result from SSc-associated fibrosis (sicca syndrome) or lymphocytic sialadenitis in the context of secondary Sjögren’s syndrome (Avouac et al. 2006). Sicca syndrome was reported in 70% of SSc patients, while secondary Sjögren’s syndrome, as defined by the American-European Consensus Group criteria, is affecting up to 14% of SSc patients and significantly associated with the lcSSc subtype (Avouac et al. 2006). Furthermore, a reduction in salivary flow, resulting in xerostomia, is primarily attributed to salivary gland fibrosis (Avouac et al. 2006). Xerostomia increases the susceptibility of SSc patients to periodontitis, tooth decay, and erosion by reducing saliva’s capacity to buffer the heightened oral acidity resulting from bacterial activity and SSc-associated gastroesophageal reflux (Almeida et al. 2023).

### Dysgeusia

Dysgeusia, a disturbance in taste sensation (Jafari et al. 2021), was reported significantly in SSc patients, manifested as perceiving gustatory stimuli as bitter, sour, or metallic (Crincoli et al. 2016). To date, no study has investigated the etiology of dysgeusia in SSc, although it may be attributed to xerostomia, SSc-associated Sjögren’s syndrome, microbial colonization in periodontal pockets, or pathological changes in gustatory nerve fibers (Jafari et al. 2021).

### Temporomandibular Joint Involvement

SSc patients exhibit restricted mandibular movements and are prone to temporomandibular disorder (TMD) and jaw pain compared to healthy individuals (Crincoli et al. 2016). These manifestations can be linked to the pathological changes in the TMJ of SSc patients, including condylar resorption, calcinosis, erosive synovitis, or synovial fibrosis (MacIntosh et al. 2015; Crincoli et al. 2016). However, while arthropathy commonly affects SSc patients, determining whether the impaired mandibular function results from TMJ involvement or fibrosis of skin, muscle, and subcutaneous tissues, or a combination of both, is challenging (Crincoli et al. 2016). Whether lsSSc and dcSSc contribute to distinct TMJ involvements and their prevalence remain uninvestigated.

### Mandibular and Facial Bone Resorption

Resorption of mandibular and facial bone in SSc is a multifactorial process influenced by SSc-associated microvasculopathy and excessive facial skin and muscle tension leading to ischemia and pressure necrosis (Crincoli et al. 2016). This resorption is particularly evident in muscle attachment areas, such as the mandibular angle, condylar head, coronoid process, and zygomatic arch (Burchfield and Vorrasi 2019). Mandibular condyle is more susceptible to resorption in SSc patients, with prevalence rates of 10% to 50%, and interestingly, cases of secondary apertognathia due to condylar resorption have been reported in SSc (MacIntosh et al. 2015).

### Trigeminal Neuropathy

Trigeminal neuralgia (TN) is more prevalent in SSc patients than in general individuals (Maltez et al. 2021). Interestingly, patients presented TN signs preceding their diagnosis of SSc, suggesting it as a presenting symptom of SSc (Maikap and Padhan 2022). TN manifestations include severe, pricking, intermittent electric shock-like pain on the side of the face, which is triggered by chewing, talking, or teeth brushing (Maikap and Padhan 2022). TN associated with SSc is poorly understood, but potential factors include neural tissue fibrosis leading to increased endoneurial pressure and damage to myelinated trigeminal fibers, vasculitic changes, or, rarely, secondary to severe mandibular resorption and compression neuropathy (Maltez et al. 2021; Maikap and Padhan 2022).

### Alterations in Oral Microbiota

Studies on oral microbiome in SSc patients are scant. However, a recent study showed significantly higher subgingival *Eikenella corrodens* levels in lcSSc patients compared to healthy controls, a correlation between *Porphyromonas gingivalis* and severe periodontitis in lcSSc, and exclusive association of *Prevotella intermedia* with specific IL-1 gene polymorphisms in lcSSc patients (Jud et al. 2023). Interestingly, tongue swabs from both SSc and healthy individuals showed notably lower *Lactobacillus* amount in the SSc group (Melchiorre et al. 2021). Conversely, *Lactobacillus* is significantly increased in the gut microbiota of SSc patients, especially in lcSSc, with a potential contribution to SSc-associated gastrointestinal involvement (Melchiorre et al. 2021). Such findings highlight the poorly understood interplay of *Lactobacillus* across oral–gut axis microbiota in SSc. Collectively, these studies are pioneering, yet limited by sample size and scope. Future research with larger SSc patient groups and varied oral samples, including biofilms, saliva, and GCF, are essential to elucidate the mechanisms driving oral microbiome alterations in SSc, considering the interconnection between oral–gut microbiota and immune dysregulation in SSc.

## Insights into the Molecular Basis of Skin Fibrosis and Periodontal Phenotype of SSc Interleukins

Naive T cells, when stimulated, differentiate into 2 T-cell subtypes: T helper cell (Th) 1 and Th2; the cytokines produced by these subtypes regulate both inflammatory responses and ECM deposition; Th1 cytokines suppress ECM deposition, yet Th2 cytokines promote it (Chizzolini et al. 2011). In SSc, there is an overproduction of the profibrotic Th2 cytokines IL-4, IL-5, IL-6, IL-10, and IL-13 (Shima 2021). Indeed, IL-6 and IL-10 exhibited elevated serum levels in SSc patients at early disease phase and correlated with extent of skin fibrosis (Matsushita et al. 2006). Although IL-10 has an anti-inflammatory effect, its increase in SSc has been suggested as a feedback loop to induce proinflammatory cytokines such as IL-6, which has a crucial role in SSc pathogenesis (Shima 2021). Tocilizumab (TCZ), an IL-6 receptor α inhibitor, alleviates the persistent fibrotic phenotype of lesional SSc dermal fibroblasts in explant culture (Denton et al. 2018) and impairs bleomycin-induced skin fibrosis (Desallais et al. 2014). Compared to heathy subjects, elevated IL-6 levels exist in the saliva of patients with periodontitis, although these levels do not correlate with periodontitis progression (Kawamoto et al. 2020). Similarly, SSc patients show elevated IL-6 levels in the GCF, but these do not correlate with any clinical parameters of periodontitis (Jung et al. 2023). Similar results were obtained in a preliminary study with a small number of dcSSc patients using subgingival biofilm samples, which researchers employed to address the challenge of limited GCF volume, particularly in healthy individuals (Buchbender et al. 2021). However, GCF, as a serum-derived fluid, offers greater sensitivity for detecting biomarkers that reflect real-time inflammatory phases within periodontal tissues than biofilm, which is more influenced by external factors and microbial activities. Hence, the extent to which GCF results correlate with those of the biofilm remains uncertain (Buchbender et al. 2021).

**Figure 2.**
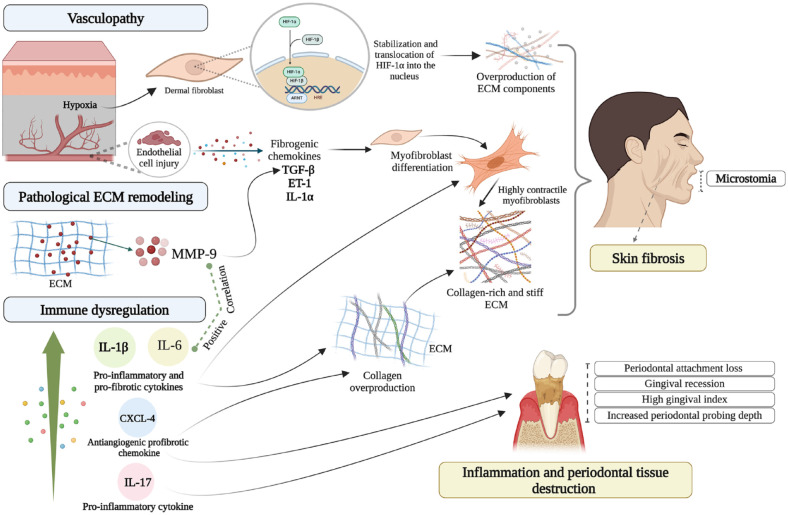
Molecular mechanisms underlying the orofacial manifestations of systemic sclerosis (SSc). Vasculopathy associated with SSc causes hypoxic microenvironment and hypoxia-inducible transcription factor 1α (HIF-1α) stabilization and translocation to the nucleus, which then induces expression of extracellular matrix (ECM) component-encoding genes. Endothelial cell injury also induces the production of the fibrogenic chemokines transforming growth factor β (TGF-β), endothelin 1 (ET-1), and interleukin (IL) 1α, which promote myofibroblasts differentiation and ECM deposition. Elevated matrix metalloproteinase 9 (MMP-9) serum levels of dcSSc patients are associated with activation of latent TGF-β and skin fibrosis. The increased level of MMP-9 in periodontal pickets of SSc patients is also associated with pathological ECM remodeling, higher level of IL-6, and periodontal tissue destruction. Immune dysregulation in SSc is manifested with overproduction of proinflammatory and profibrotic cytokines, IL-6, IL-1β, and IL-17, as well as increased CXCL-4 levels. These conditions promote fibrotic and inflammatory processes and correlate with more orofacial involvement in SSc. The figure was created with BioRender.com.

Furthermore, serum IL-6 levels increase within the early SSc stage (<3 y), promoting the inflammatory phase of SSc and vascular damage. This increase is followed by a reduction in IL-6 as SSc progresses, along with an increase in IL-13 levels on the other side, indicating the subsequent fibrotic stage of the SSc (Shima 2021). Collectively, these findings indicate that, in SSc, although IL-6 may contribute to fibrosis (Fig. 2) (Denton et al. 2018), elevated IL-6 expression in the oral cavity may simply reflect the early phase of SSc or that SSc patients show a hyperactive immune response and thus may not be a direct contributor to oral disease progression.

IL-1β has a significant role in fibrosis by stimulating collagen deposition and myofibroblast differentiation (Aden et al. 2010). Increased IL-1β levels in serum and skin biopsies from SSc patients were positively correlated with fibrotic and inflammatory skin scores (Lin et al. 2019; Laurent et al. 2022). Furthermore, IL-1β has been considered as a key modulator in promoting periodontal tissue destruction (Fig. 2) (Cheng et al. 2020). In a study of lcSSc patients, periodontitis was found in 31 of 38 patients, and in these, an IL-1β polymorphism (C3953 allele) was linked to the presence of pathogenic *P. intermedia* bacteria (Jud et al. 2023).

Th17, a T helper cell subset, produces IL-17, a proinflammatory cytokine that promotes neutrophil migration and activation and the production of IL-6 and IL-8 by gingival fibroblasts (Beklen et al. 2007; Chizzolini et al. 2011; Shima 2021). Although studies have yet to investigate the contribution of IL-17 to oral complications in SSc patients, elevated IL-17 messenger RNA (RNA) and protein expression in the lesional skin and peripheral blood mononuclear cells of SSc patients have been reported (Shima 2021). Together with previous studies showing a 6-fold increase in IL-17 immunoreactivity in gingival tissues of patients with periodontitis (Beklen et al. 2007), these observations suggest that future research investigating the potential role of IL-17 in periodontitis in SSc patients may be warranted.

### Chemokine (C-X-C motif) ligand 4 (CXCL-4)

Chemokine (C-X-C motif) ligand 4 (CXCL-4) is an antiangiogenic, profibrotic chemokine associated with vasculopathy and inflammatory cell recruitment in SSc, serving as a biomarker for assessing SSc severity (van Bon et al. 2013). The level of CXCL-4 in the GCF of SSc patients is markedly elevated compared to controls; CXCL-4 levels correlate with increased measures of periodontitis, including clinical attachment level, periodontal probing depth, and overall gingival index (Fig. 2) (Jung et al. 2023).

### Matrix Metalloproteinase-9

Matrix metalloproteinases (MMPs) are extracellular endopeptidases that play roles in pathological ECM remodeling, contributing to periodontal tissue destruction (Nagase 1997). Elevated serum levels of MMP-9 (gelatinase/type IV collagenase) are found in dcSSc patients, correlating with both serum TGF-β1 levels and degree of skin fibrosis, possibly due to its ability to activate latent TGF-β (Kim et al. 2005; Mirastschijski et al. 2010) (Fig. 2). Additionally, MMP-9 levels are increased in biofilm samples of SSc patients and positively correlate with IL-6 production (Buchbender et al. 2021). However, this study did not examine individuals with both periodontal disease and SSc. Therefore, the link between SSc and periodontitis or its severity levels, in the context of the higher MMP-9 levels in SSc subgingival biofilm samples, is not possible using these findings. A later study showed the high levels of CXCL-4 and MMP-9 in the GCF of SSc patients, which were associated with adverse periodontal parameters such as clinical attachment level, periodontal probing depth, plaque index, and gingival index (Jung et al. 2023). Intriguingly, MMP-9 and CXCL4, but not IL-6, levels significantly correlated with early-onset (<2 y) disease (Jung et al. 2023).

#### The role of skin fibrosis in the clinical complications of SSc

The degree of microstomia, which is caused by cutaneous fibrosis (Geyer and Müller-Ladner 2011), directly correlates with oral complications in SSc such as clinical attachment loss (Buchbender et al. 2021), number of missing and decayed teeth, and overall periodontal parameters (Jung et al. 2023). No correlation between skin thickness score and oral features was observed (Jung et al. 2023). These data suggest that the current methods of evaluating drugs in clinical trials, which assess skin thickness score, are not appropriate for evaluating oral complications and that, in the future, the degree of microstomia could be included as an end point in clinical trials.

#### Managing orofacial complications of SSc

Currently, there is no specific approved treatment for SSc. However, management of its orofacial complications ranges from palliative treatments to dental or surgical interventions (Alantar et al. 2011). For example, mouth exercises can improve the interincisal distance in SSc patients with microstomia (Alantar et al. 2011). However, severe microstomia may hinder prosthesis construction, necessitating commissurotomy; yet, impaired wound healing may lead to scarring and microstomia recurrence. Thus, foldable silicone prostheses may serve as an alternative (Mosaddad et al. 2023). Additionally, SSc-associated xerostomia can be managed with sialagogue treatments, including tincture of pilocarpine, anetholtrithione, or salivary spray substitute (Alantar et al. 2011). Moreover, telangiectasia in SSc is typically left untreated; while light-based treatments show improvement, they do not prevent its recurrence (Alantar et al. 2011; Veale et al. 2016). Typically, no intervention is required for PDL widening in SSc, as no clinical complications or correlations with deteriorated periodontal conditions have been reported (Jung et al. 2023). Interestingly, despite SSc patients’ elevated rates of periodontitis and impaired bone turnover, studies indicated that dental implants can be placed successfully without exacerbating overall morbidity or conflicting with systemic treatments, with the survival rate of implants unaffected by SSc, suggesting that implants can effectively enhance oral health–related quality of life by alleviating the functional and psychological impacts of SSc (Mosaddad et al. 2023). However, such successful treatments require thorough preoperative risk assessment and regular follow-up, as well as depend on SSc severity, associated vasculopathy, and bone quality (Mosaddad et al. 2023). For instance, dental implants may be contraindicated in SSc patients with severe vasculopathy (Alantar et al. 2011). Furthermore, facial and mandibular bone resorption associated with SSc has no treatment options, while routine follow-up can be beneficial to manage the exacerbation of symptoms (Alantar et al. 2011). However, surgical reconstruction of TMJ after condylar resorption showed promise in restoring functional occlusion and mandibular movements in SSc patients (MacIntosh et al. 2015). Despite the long-term success observed, the overall health of SSc patients and the severity of the disease remain critical factors in assessing the feasibility of such invasive maxillofacial surgeries.

## Conclusions and Future Directions

In a recent study of the oral cavity of SSc patients, the expression of MMP-9 and CXCL4, but not IL-6, in the GCF correlated with early-stage SSc and several parameters of periodontitis (Jung et al. 2023). These findings highlight the need for further investigations into the involvement of proinflammatory cytokines, including CXCL-4 and MMPs such as MMP-9, as mediators of oral and periodontal disease in SSc and as potential predictive biomarkers for SSc. In addition, future clinical trials could incorporate oral end points, such as the degree of microstomia. This point is important, for example, since although an anti–IL-6 antibody was recently found not to affect skin fibrosis in SSc patients (Khanna et al. 2020), such a treatment was recently shown to improve periodontal outcomes in patients with rheumatoid arthritis (Ancut,a et al. 2021). Given that oral complications of SSc can indirectly cause malnutrition and negatively affect psychological well-being (Baron et al. 2015; Türk et al. 2020), further consideration of the oral symptoms of SSc should substantially ameliorate the overall quality of life of SSc patients.

## Author Contributions

M. Sharma, contributed to conception, design, data acquisition, analysis, and interpretation, drafted and critically revised the manuscript; A. Fadl, contributed to data acquisition, analysis, and interpretation, drafted and critically revised the manuscript; A. Leask, contributed to conception, design, data acquisition, analysis, and interpretation, critically revised the manuscript. All authors gave final approval and agree to be accountable for all aspects of the work.
